# Successful Back Contouring With Elimination of Back Rolls Using Ultrasound-Assisted Liposuction and Helium-Activated Radiofrequency

**DOI:** 10.1093/asjof/ojaa036

**Published:** 2020-10-26

**Authors:** Arian Mowlavi, Armin Talle, Marian Berri, Waleed Rashid

## Abstract

**Background:**

Patients are routinely consulted regarding dislike of their upper and middle back contour and associated back rolls that stick out of their bras. Although patients only associate this fullness with excess fat, on examination it becomes evident that back rolls are due to a combination of excess fat as well as skin redundancy. To date, treatment of both excess skin and fat in back rolls has required consideration of excisional surgery such as an upper body lift.

**Objectives:**

We present 14 consecutive back contouring cases that were treated with an alternative protocol involving simultaneous ultrasound assisted liposuction and helium activated radiofrequency.

**Methods:**

Patients underwent ultrasound assisted liposuction to remove superficial fat over the upper and middle back as well as helium activated radiofrequency to tighten the skin using subdermal coagulation.

**Results:**

All 14 patients visually demonstrated elimination of back rolls and improvement in upper and middle back contour. All 14 patients also reported overall satisfaction in their postoperative follow-ups at 3, 6, and 12-months.

**Conclusion:**

In summary, simultaneous ultrasound assisted liposuction and helium activated radiofrequency provide an effective treatment for patients desiring improvements in upper and middle back contour and elimination of back rolls while avoiding more invasive excisional surgeries.

**Level of Evidence: 4:**

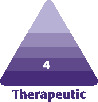

Patients are routinely consulted in our office regarding improving the contour of their upper and middle back. Patients are most concerned about back rolls that fall out of their bras and which they associate with being overweight. As evident by physical examination findings, patients are educated that back rolls are associated with not only excess fat but also more importantly redundant skin.^1^ Additionally, there is a zone of fibrous adhesion just under these areas of fat that results in the characteristic appearance. Previous attempts to eliminate back rolls have not been successful for 2 reasons. First, the fat in this region is often predominately superficial and not amenable to optimal removal using traditional liposuction techniques.^2^ This is because traditional liposuction cannulae can only remove deep fat that is loose and not superficial fat that maintains retaining ligaments that prevent uniform cannula penetration. In addition, back rolls maintain a dominant component of skin redundancy.^3^ Traditionally, elimination of skin redundancy has often necessitated excisional surgery such as an upper body lift that has the stigma of an unsightly surgical incision and subsequent scar.^4-6^

We present a novel and effective approach to back contouring with the elimination of back rolls that involves the use of ultrasound-assisted liposuction (UAL) and helium-activated radiofrequency (RF). The UAL allows for emulsification of fat from the superficial layer, which then is amenable to removal with liposuction cannulae that traverse in the deep fat layer through compression coupling techniques.^7^ In the process of fat removal from the superficial layer, a plane is created below the subdermal plexus. This plane is then accessible by the helium-activated RF-emitting probe that allows for skin tightening by a process termed subdermal coagulation.^8-10^ We present 14 consecutive patients treated for back contouring with the elimination of back rolls using the above technique.

## METHODS

We present 14 consecutive patients over 2 years (January 2016 to January 2018) treated for the dislike of their back contour including back rolls. Retrospective data were ethically evaluated using guiding principles from the Declaration of Helsinki. Each patient signed an informed consent to surgery and the use of data retrospectively. All 14 patients presented with minimal to moderate skin redundancy in a preoperative exam. Patients complained that the rolls were not only unsightly but also often caused them discomfort when they fell out of their bras. Eleven of the patients presented with 1 back roll, 2 patients presented with 2 back rolls, and 2 patients presented with 3 back rolls. Patients with severe skin redundancy require surgical excision of skin and were excluded from the study. All patients were placed in the prone position and underwent UAL (VASER(R), Raleigh, NC, USA) of their upper and middle back. Infiltration and aspiration volumes ranged between 100 cc and 300 cc per side (specific values listed in figure legends). VASER settings were uniform across all patients: 3.3 mm 5-ringed probe at 80% power. All patients were then treated simultaneously with helium-activated RF energy (RENUVION (R), Clearwater, FL, USA) at 80% power and 3 L/minute of helium flow to administer subdermal coagulation. Treatment was provided using probes that extended throughout the upper and middle back using the same liposuction portholes. Patients received subdermal coagulation with 6 passes of 80% power and 3 L/minute of helium flow. All portholes were repaired with simple skin stitches using 5-0 fast-absorbing suture. Patients were placed in customized compression garments with foam inlays, which were worn for 2 weeks. Patients received 5 lymphatic messages performed on days 2, 4, 6, 8, and 10 following surgery.

## RESULTS

**Figure 1.**
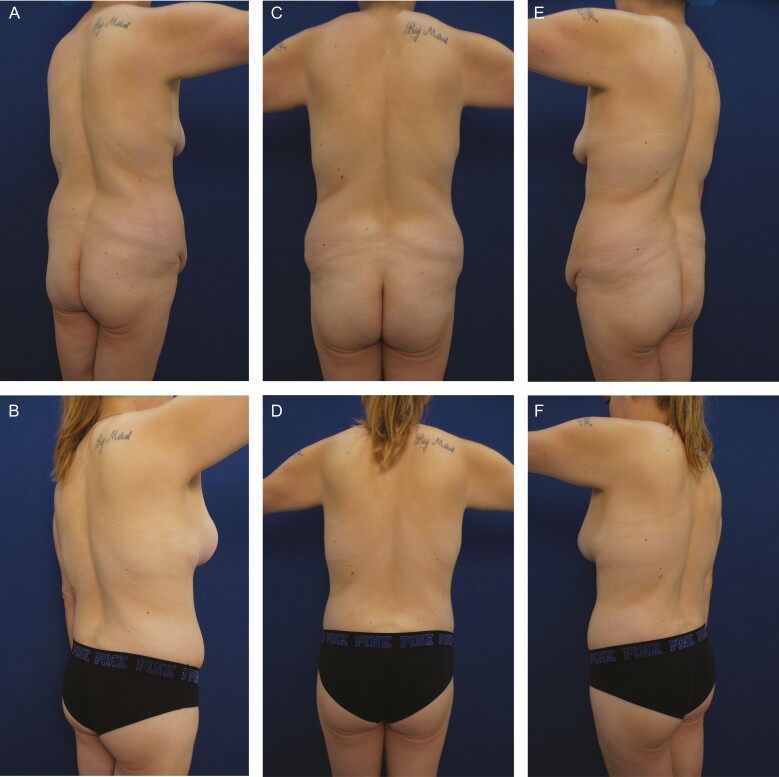
Elimination of back rolls following ultrasound-assisted liposuction and helium-activated radiofrequency treatment. This 35-year-old female patient demonstrates skin redundancy and excess fat, which led to the formation of a mid-back roll. Successful treatment required liposuction with infiltration and aspiration of 100 cc tumescent solution per side as well as skin tightening using subdermal coagulation. Photographs were taken before and 3 months after surgery. Preoperative and 3 months postoperative (A, B) right lateral view, (C, D) posterior view, and (E, F) left lateral view.

**Figure 2.**
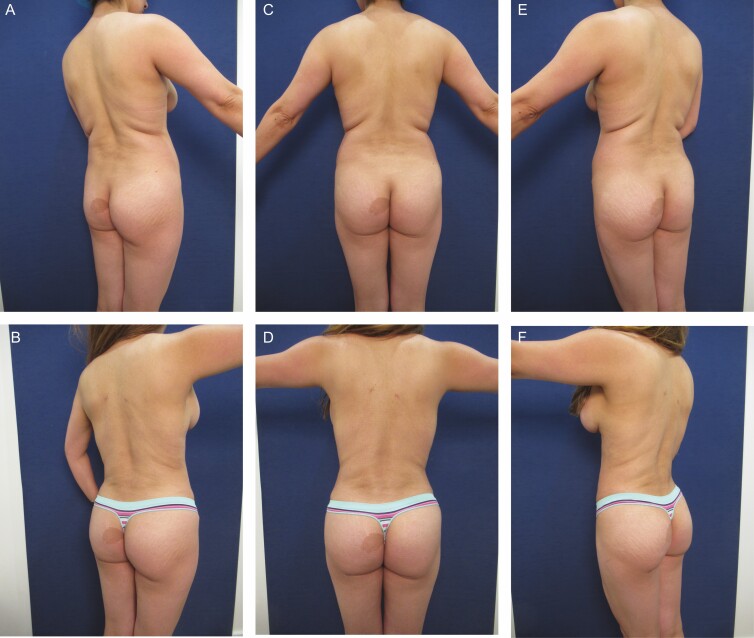
Elimination of back roll following ultrasound-assisted liposuction and helium-activated radiofrequency treatment. This 44-year-old female patient demonstrates skin redundancy and excess fat, which led to the formation of a mid-back roll. Successful treatment required liposuction with infiltration and aspiration of 100 cc tumescent solution per side as well as skin tightening using subdermal coagulation. Photographs were taken before and 3 months after surgery. Preoperative and 3 months postoperative (A, B) right lateral view, (C, D) posterior view, and (E, F) left lateral view.

**Figure 3.**
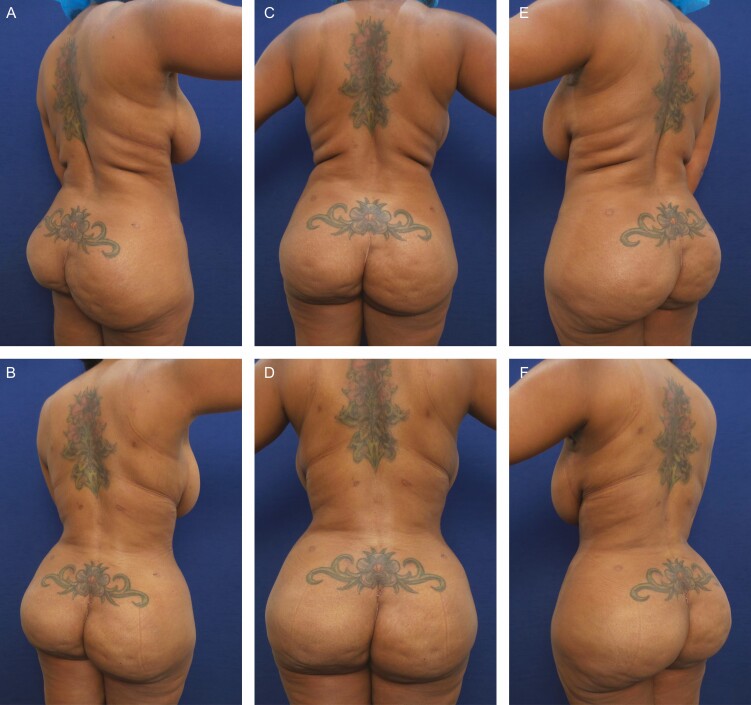
Elimination of back roll following ultrasound-assisted liposuction and helium-activated radiofrequency treatment. This 47-year-old female patient presented excessive skin redundancy, leading to the formation of 3 back rolls as pictured above. Successful treatment required liposuction with infiltration and aspiration of 150 cc tumescent solution per side as well as skin tightening using subdermal coagulation. Photographs were taken before and 3 months after surgery. Preoperative and 3 months postoperative (A, B) right lateral view, (C, D) posterior view, and (E, F) left lateral view.

**Figure 4.**
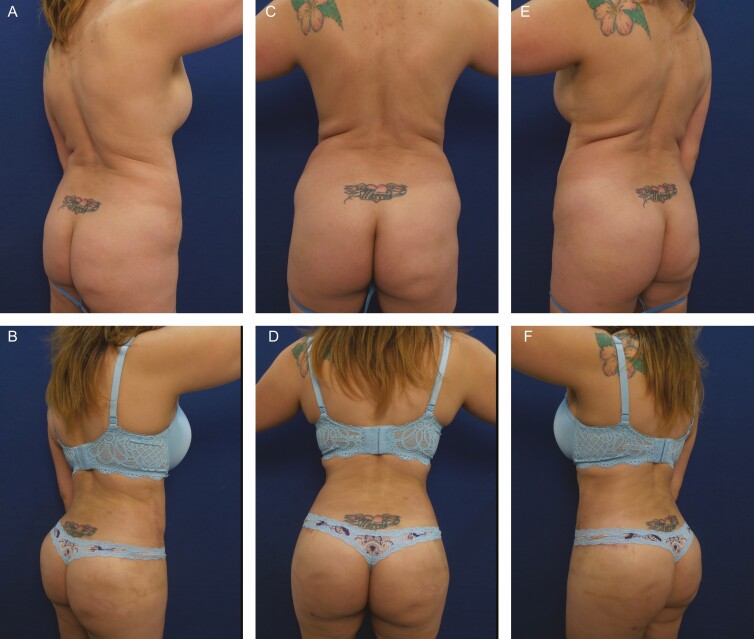
Elimination of back roll following ultrasound-assisted liposuction and helium-activated radiofrequency treatment. This 48-year-old female patient demonstrates skin redundancy and excess fat, which led to the formation of a back roll. Successful treatment required liposuction with infiltration and aspiration of 250 cc tumescent solution per side as well as skin tightening using subdermal coagulation. Photographs were taken before and 3 months after surgery. Preoperative and 3 months postoperative (A, B) right lateral view, (C, D) posterior view, and (E, F) left lateral view.

**Figure 5.**
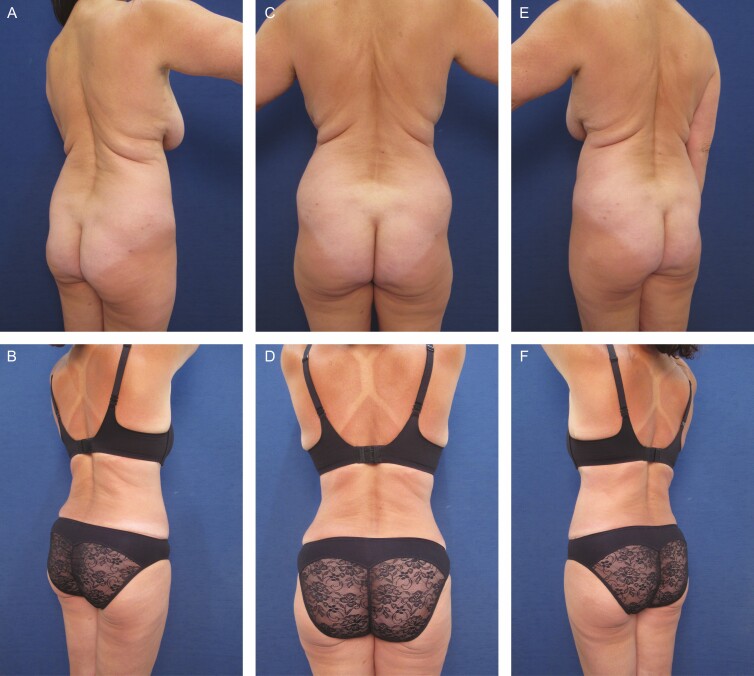
Elimination of back roll following ultrasound-assisted liposuction and helium-activated radiofrequency treatment. This 61-year-old female patient presented excess fat and skin redundancy, which led to the formation of 2 back rolls. Successful treatment required liposuction with infiltration and aspiration of 200 cc tumescent solution per side as well as skin tightening using subdermal coagulation. Photographs were taken before and 3 months after surgery. Preoperative and 3 months postoperative (A, B) right lateral view, (C, D) posterior view, and (E, F) left lateral view.

**Figure 6.**
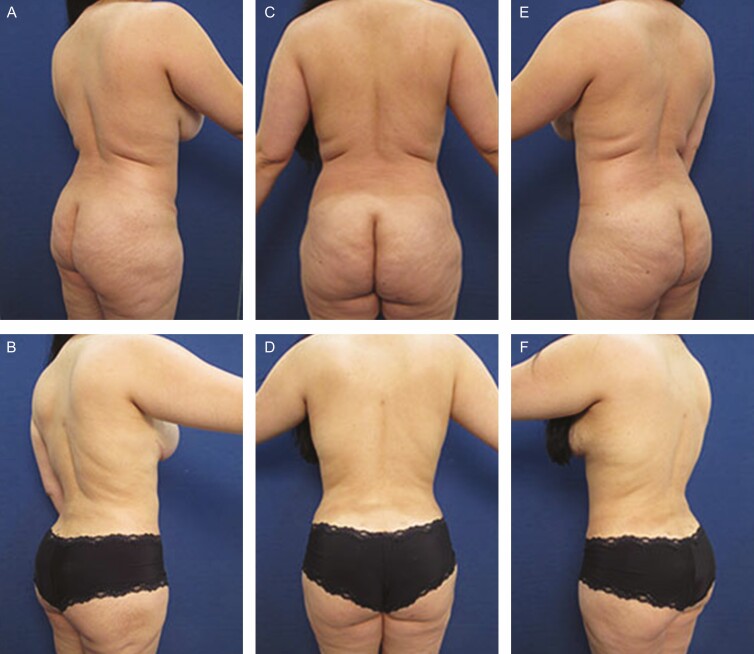
Elimination of back roll following ultrasound-assisted liposuction and helium-activated radiofrequency treatment. This 39-year-old female patient presented with 2 back rolls due to excessive fat and skin redundancy. Successful treatment required liposuction with infiltration and aspiration of 200 cc tumescent solution per side as well as skin tightening using subdermal coagulation. Photographs were taken before and 3 months after surgery. Preoperative and 3 months postoperative (A, B) right lateral view, (C, D) posterior view, and (E, F) left lateral view.

**Figure 7.**
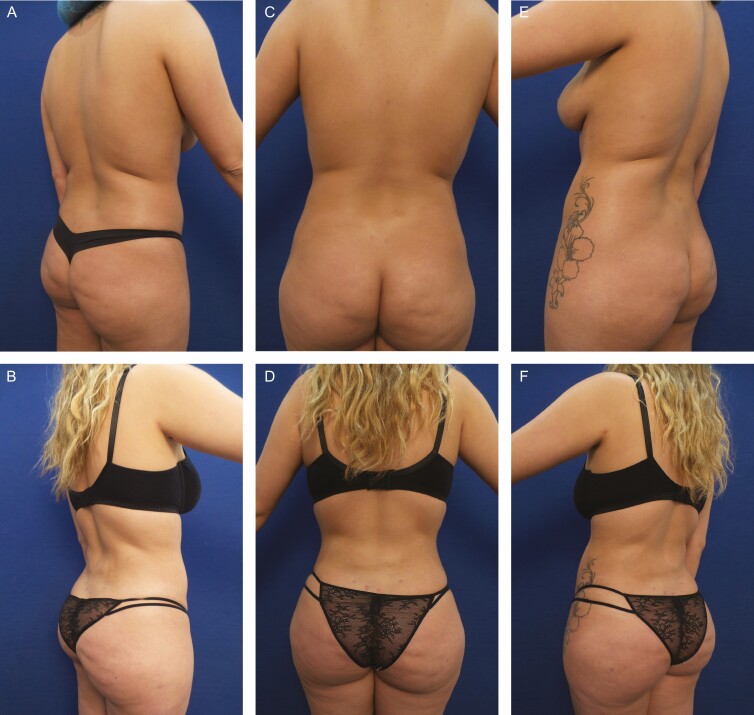
Elimination of back roll following ultrasound-assisted liposuction and helium-activated radiofrequency treatment. This 21-year-old female patient had skin redundancy and excess fat, leading to the formation of 1 back roll. Successful treatment required liposuction with infiltration and aspiration of 300 cc tumescent solution per side as well as skin tightening using subdermal coagulation. Photographs were taken before and 3 months after surgery. Preoperative and 3 months postoperative (A, B) right lateral view, (C, D) posterior view, and (E, F) left lateral view.

**Figure 8.**
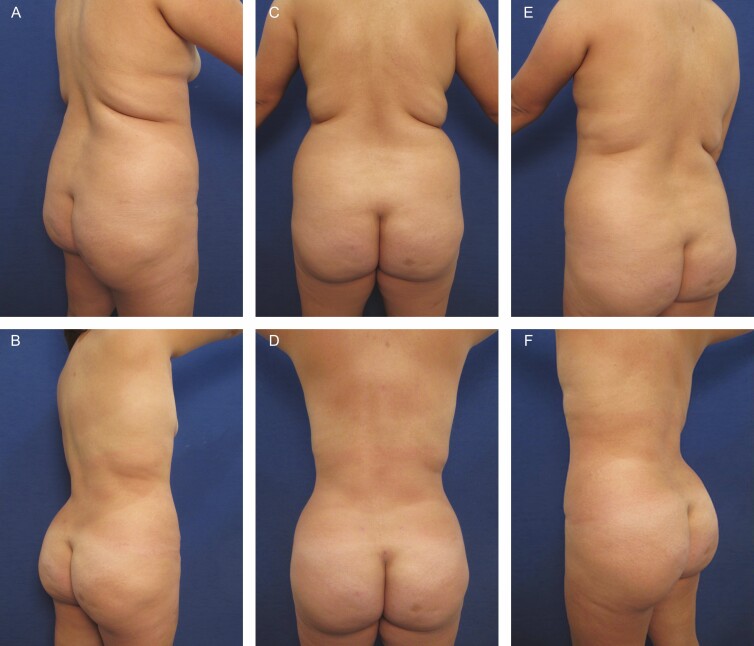
Elimination of back roll following ultrasound-assisted liposuction and helium-activated radiofrequency treatment. The 26-year-old female presented with excessive fat and middle back skin redundancy, leading to the formation of 1 back roll. Successful treatment required liposuction with infiltration and aspiration of 300 cc tumescent solution per side as well as skin tightening using subdermal coagulation. Photographs were taken before and 3 months after surgery. Preoperative and 3 months postoperative (A, B) right lateral view, (C, D) posterior view, and (E, F) left lateral view.

**Figure 9.**
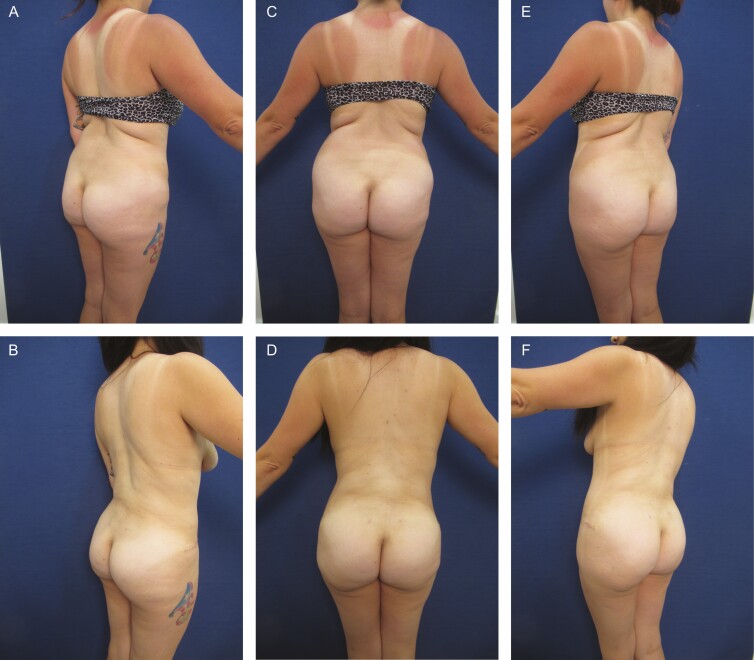
Elimination of back roll following ultrasound-assisted liposuction and helium-activated radiofrequency treatment. The 35-year-old female presented with excessive middle back skin redundancy, leading to the formation of a back roll. Successful treatment required liposuction with infiltration and aspiration of 200 cc tumescent solution per side as well as skin tightening using subdermal coagulation. Photographs were taken before and 3 months after surgery. Preoperative and 3 months postoperative (A, B) right lateral view, (C, D) posterior view, and (E, F) left lateral view.

**Figure 10.**
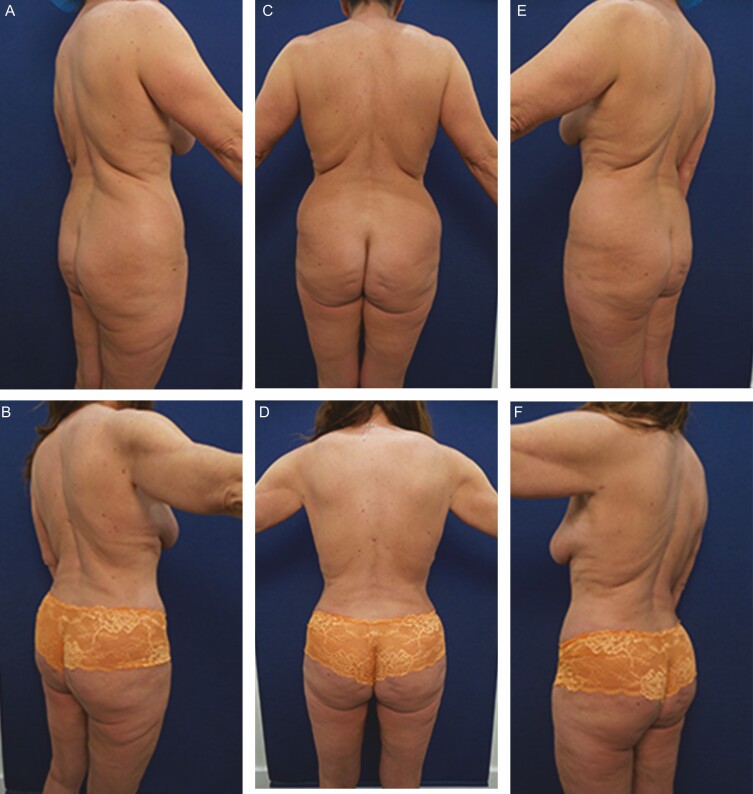
Elimination of back roll following ultrasound-assisted liposuction and helium-activated radiofrequency treatment. This 51-year-old female patient demonstrates excessive fat and skin redundancy, leading to the formation of 1 back roll. Successful treatment required liposuction with infiltration and aspiration of 250 cc tumescent solution per side as well as skin tightening using subdermal coagulation. Photographs were taken before and 3 months after surgery. Preoperative and 3 months postoperative (A, B) right lateral view, (C, D) posterior view, and (E, F) left lateral view.

**Figure 11.**
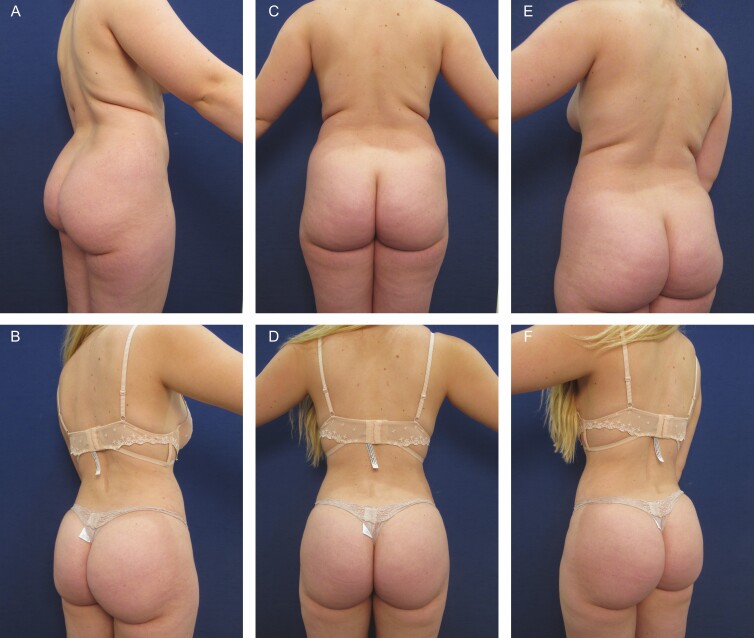
Elimination of back roll following ultrasound-assisted liposuction and helium-activated radiofrequency treatment. The 23-year-old female patient presented with excessive middle back skin redundancy, leading to the formation of 1 back roll. Successful treatment required liposuction with infiltration and aspiration of 300 cc tumescent solution per side as well as skin tightening using subdermal coagulation. Photographs were taken before and 3 months after surgery. Preoperative and 3 months postoperative (A, B) right lateral view, (C, D) posterior view, and (E, F) left lateral view.

**Figure 12.**
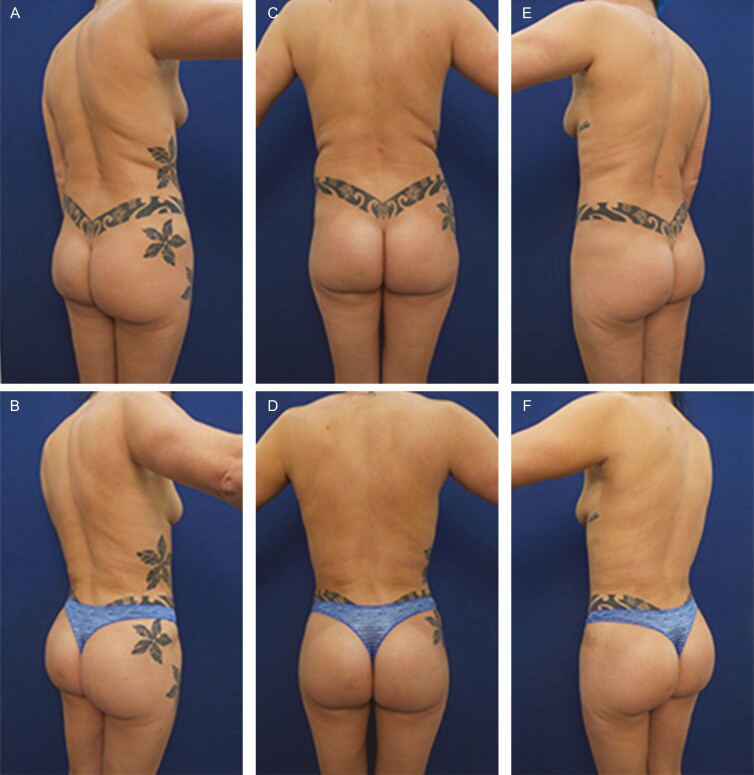
Elimination of back roll following ultrasound-assisted liposuction and helium-activated radiofrequency treatment. This 30-year-old female patient presented with 2 back rolls due to excessive fat and skin redundancy. Successful treatment required liposuction with infiltration and aspiration of 300 cc tumescent solution per side as well as skin tightening using subdermal coagulation. Photographs were taken before and 3 months after surgery. Preoperative and 3 months postoperative (A, B) right lateral view, (C, D) posterior view, and (E, F) left lateral view.

**Figure 13.**
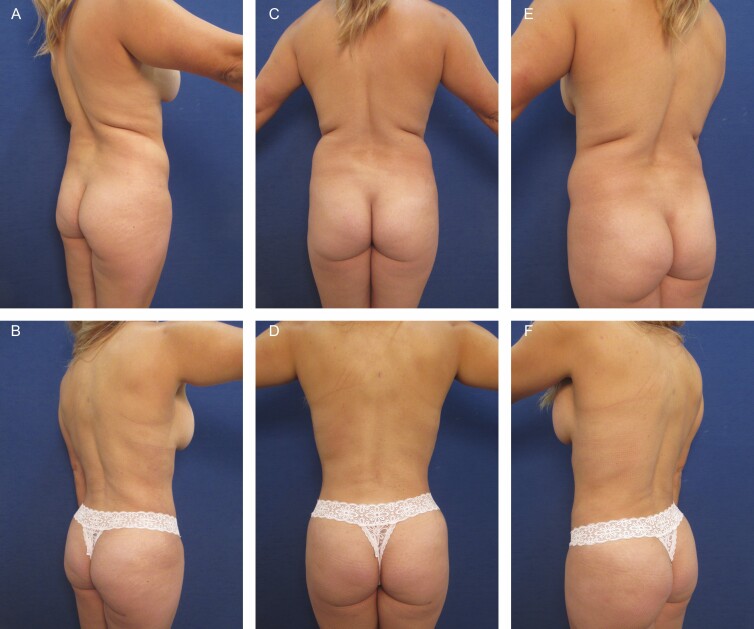
Elimination of back roll following ultrasound-assisted liposuction and helium-activated radiofrequency treatment. This 40-year-old female patient presented with excessive middle back skin redundancy, leading to the formation of 1 back roll. Successful treatment required liposuction with infiltration and aspiration of 250 cc tumescent solution per side as well as skin tightening using subdermal coagulation. Photographs were taken before and 3 months after surgery. Preoperative and 3 months postoperative (A, B) right lateral view, (C, D) posterior view, and (E, F) left lateral view.

**Figure 14.**
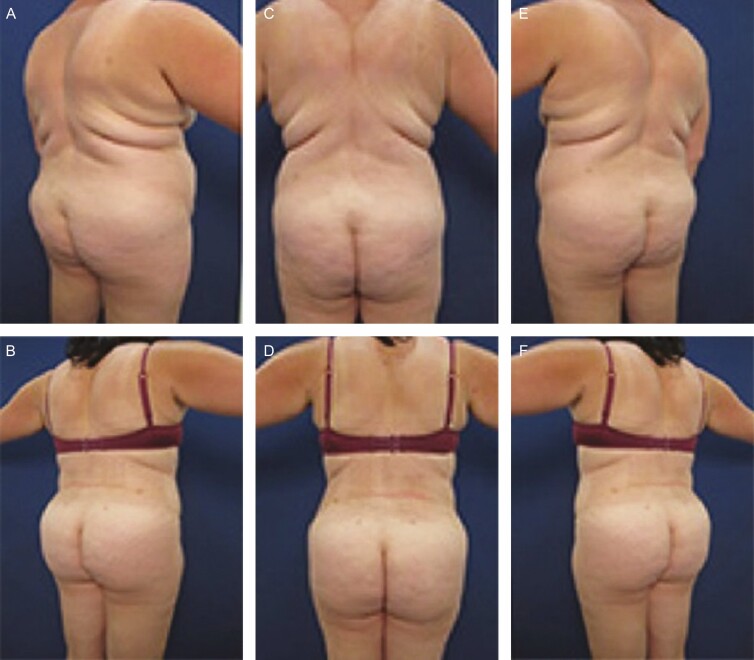
Elimination of back roll following ultrasound-assisted liposuction and helium-activated radiofrequency treatment. This 50-year-old patient presented with excessive middle back skin redundancy, leading to the formation of 3 back rolls. Successful treatment required liposuction with infiltration and aspiration of 250 cc tumescent solution per side as well as skin tightening using subdermal coagulation. Photographs were taken before and 3 months after surgery. Preoperative and 3 months postoperative (A, B) right lateral view, (C, D) posterior view, and (E, F) left lateral view.

Patients were all females and with an average age of 39 years (range, 21-61 years). All patients demonstrated the elimination of their back rolls with improved back contour as depicted by the creation of a “~” tilde curve. Patients were evaluated with subjective satisfaction based on postoperative visit notes and postoperative photographs at 6 months follow-up. There were no noted complications. All patients were satisfied by their aesthetic result and resolution of discomfort associated with preoperative back rolls falling out of their bras. Patient before and after photographs are shown in Figures 1-14.

## DISCUSSION

When presented with patients of back contouring, the traditional options often involve fat removal modalities or excisional surgeries. However, while excisions result in an unwanted scar, fat reduction modalities may ignore a critical factor to the irregularities, excess skin. These procedures may also be suboptimal because the majority of fat in the upper and middle back are limited to the superficial layer that is not amenable to optimal removal with traditional liposuction techniques. Instead, UAL technology is able to effectively remove the superficial fat in these areas by first emulsifying this fat that is intimately associated with superficial retaining ligaments. Furthermore, UAL results in a partial release of the zone of adhesion, which allows for the smooth final appearance (Figures 1-14). In addition, no viable techniques were available to address skin looseness without an excisional procedure requiring an incision line. While there are techniques such as the bra line back lift,^1^ these techniques of excisional skin removal have not been favorable as most patients who have visible back rolls, but minimal to moderate skin redundancy would like to avoid the stigma of an incision line and subsequent scarring over the upper and middle back. In this paper, we present a novel approach of treatment of upper and middle back rolls that involve excess superficial fat and skin redundancy. In fact, skin redundancy may be a bigger component than excess fat for most patients with back roll concerns. This concern can now be addressed using minimally invasive helium-activated RF subdermal coagulation skin tightening. The necessity of the RF skin tightening varied from patient to patient, but it did result in a tighter, leaner appearance in all 14 patients. While UAL allows for increased skin retraction compared to traditional liposuction modalities, it is still often not enough for the complete elimination of fat and skin redundancy because the reduction of fat following UAL results in greater skin redundancy compared to preoperative measurements. For this reason, we found that the helium-activated RF skin tightening a necessary component in these results. However, as this study primarily deals with patients of minimal to moderate skin redundancy, further research must be done to determine the back skin retraction capacity of UAL and RF subdermal coagulation. Additionally, while all 14 patients showed a clear elimination of visible back rolls, we will pursue additional research with objective measurements in our postoperative analysis to further determine the efficacy of this modality.

## CONCLUSIONS

We presented 14 successful elimination of back rolls and aesthetic back contouring using UAL and helium-activated RF treatments that are less invasive than a traditional upper body lift. All patients treated demonstrated aesthetic improvement of back contour and elimination of back rolls. Patients were followed up at 6 months with postoperative documentation of back roll elimination and subjective satisfaction of their results. In summary, we present an effective alternative to prior contouring procedures that benefit from avoidance of surgical incision lines, unsightly scars, and more invasive maneuvers.
